# Aromatic Amino Acids-Guanidinium Complexes through Cation-π Interactions

**DOI:** 10.3390/molecules20059214

**Published:** 2015-05-20

**Authors:** Cristina Trujillo, Ana A. Rodriguez-Sanz, Isabel Rozas

**Affiliations:** 1 Trinity Biomedical Sciences Institute, School of Chemistry, Trinity College Dublin, 152-160 Pearse Street, Dublin 2, Ireland; E-Mail: trujillc@tcd.ie; 2 Departamento de Química Física, Facultade de Ciencias, Universidade de Santiago de Compostela, Campus de Lugo, Avda. Alfonso X El Sabio s/n, 27002 Lugo, Spain; E-Mail: anaangustias.rodriguez@usc.es

**Keywords:** guanidinium cation, aromatic amino acids, cation-π interactions, hydrogen bond, non-covalent interactions, aromaticity

## Abstract

Continuing with our interest in the guanidinium group and the different interactions than can establish, we have carried out a theoretical study of the complexes formed by this cation and the aromatic amino acids (phenylalanine, histidine, tryptophan and tyrosine) using DFT methods and PCM-water solvation. Both hydrogen bonds and cation-π interactions have been found upon complexation. These interactions have been characterized by means of the analysis of the molecular electron density using the Atoms-in-Molecules approach as well as the orbital interactions using the Natural Bond Orbital methodology. Finally, the effect that the cation-π and hydrogen bond interactions exert on the aromaticity of the corresponding amino acids has been evaluated by calculating the theoretical NICS values, finding that the aromatic character was not heavily modified upon complexation.

## 1. Introduction

Cation-π interactions have been the objective of a vast number of experimental and computational studies since Kerbarle’s seminal publication in 1981 [1]. A very important contribution to this topic has been made by Dougherty and co-workers who showed, for example, that even in water phenyl hosts bind to cationic guests stronger than to neutral or charged molecules [2]. Moreover, they carried out a protein database assessment showing that cation-stabilization is fundamental in protein structure and function and that arginine (Arg) in particular is the residue that most often [3] binds. In addition, they also reported the importance of these interactions for protein engineering [4,5]. During the 1990s, Thornton and Singh [6] analyzed a large number of crystal structures and found that aromatic amino acids prefer stacking interactions to hydrogen bonding [7].

In 2011, Frontera *et al.* published a review on cation-π interactions analyzing the forces involved in these contacts and found that some physical properties of the aromatic systems and interacting ions are directly related to the strength of the interaction [8]. Furthermore, in 2011, this same group published that π-π interactions are influenced by the presence or absence of hydrogen bonds (HBs) that are formed in a third aromatic system far from the stacking interaction analyzed [9]. In a recent article, this group have revisited the controversial proposal that substituent effects in cation-π interactions can be attributed mainly to electrostatic effects by analyzing 171 aromatic systems interacting with Na^+^; they found that both electrostatic and π-polarization effects describe cation-π interactions [10].

Gromiha and co-workers lleagues carried out several studies on cation-π interactions responsible of protein stability. They established that the roles of cation-π interactions are different from those of other non-covalent contacts in the stability of protein structures and that Arg is more likely to form cation-π interactions than lysine (Lys) [11]. Already in 1986, Burley and Patsko demonstrated that side-chain amino groups interact with aromatic side chains by analyzing 33 protein crystal structures; they found that positively charged amino groups of Lys or Arg are preferentially located over the ring centroids of aromatic amino acids [12]. Another similar study is that published by Karlin *et al.* where they found that this type of interactions could have implications in protein folding [13].

During the last 10 years, we have worked on the design, synthesis and biological evaluation of guanidinium derivatives some of which aim to target DNA; for that reason, we previously studied the complexes established by this cation and the four DNA heteroaromatic bases [14]. We proved that all of these interactions are deeply affected by the environment and, hence, to consider aqueous solvation of guanidinium is essential for a good description of its experimental/biological properties. Considering that different families of our guanidine derivatives are aimed to interact with proteins such as receptors (α_2_-adrenoceptors) [15] or enzymes (kinases) [16] and to continue with our interest [17,18,19,20] in the interactions and properties of the guanidinium cation, we have now carried out the theoretical study of the complexes formed by this particular cation and aromatic amino acids (phenylalanine –Phe-, tyrosine –Tyr-, tryptophan –Trp- and histidine –His-).

In this particular area, the recent work of Cabaleiro-Lago and Rodriguez-Otero deserves special attention. On the one hand, they have studied the interaction of microhydrated guanidinium with the aromatic systems existing in the aromatic amino acids and found that the presence of a small number of water molecules significantly affects the characteristics of the complexes. Hydrogen bonds formed by water with the cation, another water molecule, or the aromatic units lead to a large number of minima similar in energy but very different structurally. They found that the differences in stability were mainly a consequence of the different strength of the cation···π contact [21]. On the other hand, they have recently published the study of the interaction of guanidinium with Phe, Tyr and Trp in the gas phase as neutral systems finding that the most stable minima correspond to folded amino acids, with the cation interacting simultaneously with the carboxyl oxygen, the amino nitrogen and the aromatic ring, whereas zwitterionic amino acids are as stable as neutral ones [22].

In the present study, we have chosen to use bulk solvent instead of microhydration for coherence with our previous studies. In addition to the study of the corresponding complexes, we have carried out an evaluation of the aromaticity changes induced in the aromatic rings upon complexation by calculating the corresponding nucleus-independent chemical shift NICS indexes [23]. This study has allowed us to better understand the potential interactions that guanidinium derivatives can establish when targeting proteins, which can determined their biological activity and influence their molecular design.

## 2. Results and Discussion

### 2.1. Structure and Energy

**Figure 1:**
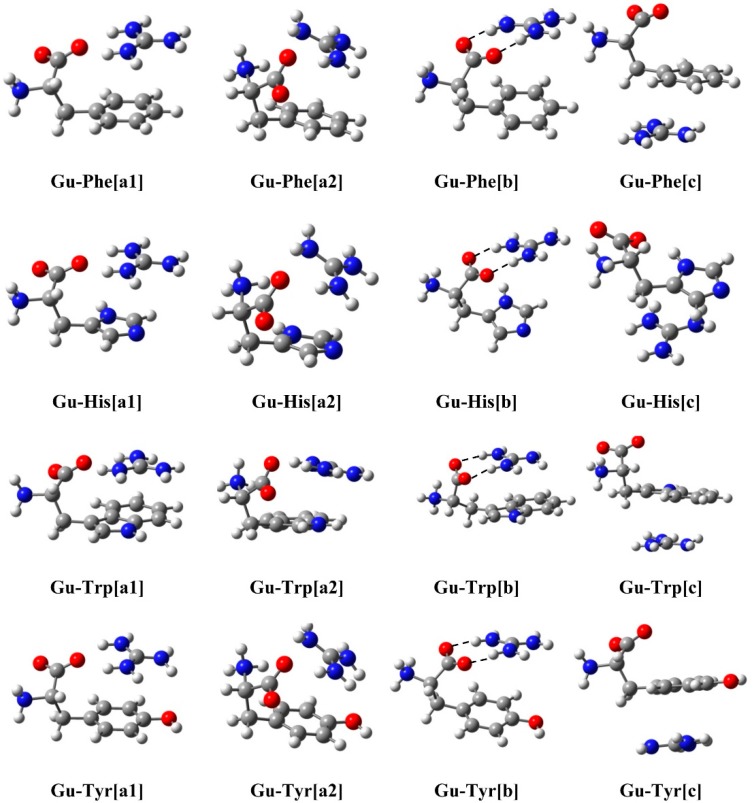
Optimized geometry of the complexes studied at the M06-2X/6-311++G(d,p) level.

We have studied all the complexes formed by the interaction between the guanidium cation and four aromatic amino acids: **Phe**, **His**, **Trp** and **Tyr**, using the M06-2X [24] DFT method at the 6-311++G(d,p) [25] computational level including water solvation by means of the SCFR-PCM approach [26]. The optimized structures are presented in Figure 1.

In all the complexes both HBs and cation-π interactions have been found. Three types of complexes have been established depending on the interactions encountered within. Complexes type **[a]** which are formed by a bifurcated HB (between two guanidinium H atoms and one O atom of the carboxylic group belonging to the amino acid) plus an additional cation-π interaction. Two different orientations can be distinguished in this type of **[a]** complexes: in the **[a1]** conformation the NH_2_ group of the amino acid is oriented backward with respect to the guanidinium, while in the **[a2]** complexes the NH_2_ group is oriented towards the cation. Type **[b]** complexes are formed by a parallel HB interaction of two guanidinium H atoms with two O atoms of the amino acid carboxylic group and also a cation-π interaction (see Figure 1 complexes **[b]**). Finally, type **[c]** complexes are formed only by cation-π interactions between the guanidinium moiety and the π cloud of the aromatic ring of the respective amino acid.

Interaction energies (E_i_, kJ·mol^−1^) are gathered in Table 1. For all amino acids, complexes of type **[a]** and **[b]**, with both HBs and cation-π interactions, are quite more stable than complexes type **[c]**, which are formed only by cation-π interactions. In all cases complexes **[a1]** with bifurcated HBs are the most stable, followed by complexes type **[b]** which present double HBs. Both kind of complexes, **[a1]** and **[b]** possess very close E_i_ values with only 2–3 kJ·mol^−1^ of difference. The only exception appears in the case of **Trp**, in which complex type **[a2]** is slightly more stable than complex **[b]**.

**Table 1:** Interaction energies (E_i_, kJ·mol^−1^) for all the complexes studied at the M06-2X/6-311++G(d,p) computational level.

M06-2x	Gu-Phe	Gu-His	Gu-Trp	Gu-Tyr
**[a1]**	−68.2	−50.3	−65.2	−69.5
**[a2]**	−60.8	−41.1	−60.1	−64.3
**[b]**	−65.6	−48.1	−55.7	−66.8
**[c]**	−21.4	−21.4	−15.9	−19.8

It is important to note that, looking at the interaction energy values for the different complexes, those with the larger E_i_ values (most stable) correspond to the complexes formed with **Tyr** and **Phe**, while the least stable complexes correspond to the guanidinium-**His** complexes. Even though the main contribution to the stability of these complexes should arise from the HBs established, **His** is considered to be the least aromatic of the four amino acids here considered and this could be the reason of the weaker interaction observed with guanidinium.

### 2.2. Analysis of the Electron Density: AIM Analysis

**Figure 2:**
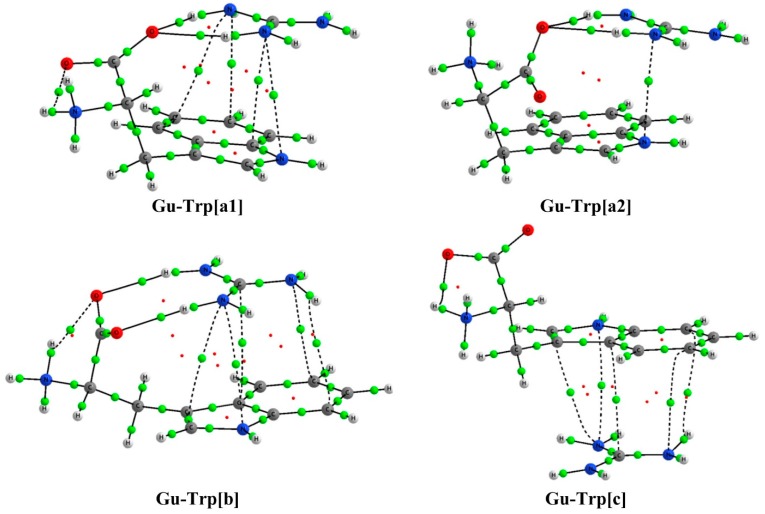
AIM-Molecular graphs of the **Gu-Trp** complexes calculated at the M06-2X/6-311++G(d,p) computational level in PCM−water. Green and red balls indicate bond and ring critical points, respectively.

The topological analysis of the electron density of the guanidinium complexes obtained, using the AIM approach [27], indicates that a number of interactions are established with the four amino acids as shown by the bond critical points (BCP) detected in the graphical analysis [28]; some examples of these graphs are exhibited in Figure 2 and the rest are presented in the Supporting Information (Figure S1). The BCPs, both for HBs and cation-π interactions, present small values of the electron density (ρ_BCP_) and positive Laplacian values (∇^2^ρ_BCP_), as shown in Table 2, indicating the closed shell characteristics of the weak interactions established among the guanidinium and the amino acids. In general, HBs (N···H or O···H) show larger ρ_BCP_ values (10^−2^ a.u. order of magnitude) than those found in the cation-π interactions (10^−3^ a.u. order of magnitude) corresponding to the relative strength of these type of contacts.

As shown in Table 2, the largest values of the ρ_BCP_ correspond to parallel HBs, which are those present in complexes type **[b]**. This is in agreement with the fact that in the case of multiple HB systems, where parallel and bifurcated complexes can be compared, parallel HB interactions are more stable and preferred over the bifurcated ones [29]. Moreover, in the present study, the largest number of cation-π contributions is found for the complexes established with **Trp**, which is the only amino acid with a bicyclic structure (indole functional group). In contrast, when the complexation occurs with **His**, BCPs associated to cation-π interactions are not found for the complexes type **[a2]** and **[c]**.

Amongst the different interactions established within these complexes (O^…^H, C^…^C, C^…^N, N^…^N) we have found an exponential relationship between the interatomic distances (in Å) and the density in the BCP for the HBs (ρ_BCP_ = 1.608 e^−2.144(d)^, see Supporting Information Figure S2) with a good R^2^ correlation coefficient of 0.972. Moreover, an exponential relationship between the Laplacian of the electron density at the BCPs for cation-π interactions and the interatomic distances (Å) has been found (∇^2^ ρ_BCP_ = 4.698 e^−1.616(d)^, see Supporting Information Figure S3), with a R^2^ coefficient of 0.804. These types of correlations have been found in previous studies with different HBs [30,31,32,33,34].

**Table 2:** AIM analysis (interaction type, ρ_BCP_ and (∇^2^ρ_BCP_, a.u.) of all the complexes studied at the M06-2X/6-311++G(d,p) computational level in PCM-water.

Complex	Type	ρ_BCP_	∇^2^ρ_BCP_	Complex	Type	ρ_BCP_	∇^2^ρ_BCP_
**Gu-Phe[a1]**	cation-π	0.0072	0.0212	**Gu-Phe[a2]**	cation-π	0.0053	0.0152
		0.0072	0.0214			0.0083	0.0254
	O1^…^H1	0.0303	0.1204		O1^…^H1	0.0283	0.1119
	O1^…^H2	0.0270	0.1130		O1^…^H2	0.0220	0.0913
**Gu-Phe[b]**	cation-π	0.0053	0.0140	**Gu-Phe[c]**	cation-π	0.0064	0.0193
		0.0059	0.0173			0.0071	0.0229
	O1^…^H1	0.0372	0.1292		N^…^H	0.0047	0.0144
	O2^…^H2	0.0322	0.1149				
**Gu-His[a1]**	cation-π	0.0062	0.0215	**Gu-His[a2]**	O1^…^H1	0.0270	0.1063
		0.0088	0.0286		O1^…^H2	0.0224	0.0940
	O^…^N	0.0091	0.0295		N^…^H	0.0090	0.0296
	O1^…^H1	0.0299	0.1173			0.0125	0.0420
	O1^…^H2	0.0266	0.1141				
**Gu-His[b]**	cation-π	0.0041	0.0119	**Gu-His[c]**	N^…^H	0.0051	0.0148
	N^…^H	0.0086	0.0264			0.0100	0.0337
	O1^…^H1	0.0374	0.1280				
	O2^…^H2	0.0350	0.1228				
**Gu-Trp[a1]**	cation-π	0.0064	0.0197	**Gu-Trp[a2]**	cation-π	0.00841	0.02968
		0.0069	0.0204		O1^…^H1	0.02628	0.09790
		0.0074	0.0223		O1^…^H2	0.02600	0.10560
		0.0078	0.0280				
	O1^…^H1	0.0305	0.1181				
	O1^…^H2	0.0256	0.1075				
**Gu-Trp[b]**	cation-π	0.00622	0.01993	**Gu-Trp[c]**	cation-π	0.00689	0.01947
		0.00635	0.01843			0.00704	0.02092
		0.00661	0.01901			0.00725	0.02356
		0.00702	0.02466			0.00725	0.02218
		0.00715	0.02102				
	O1^…^H1	0.03038	0.11460				
	O2^…^H2	0.02866	0.10782				
**Gu-Tyr[a1]**	cation-π	0.00847	0.02842	**Gu-Tyr[a2]**	cation-π	0.00740	0.02233
	O^…^H	0.00786	0.02664			0.00858	0.02565
	O1^…^H1	0.02814	0.11408		O^…^H	0.01173	0.04084
	O1^…^H2	0.02953	0.11944		O1^…^H1	0.02958	0.07545
					O1^…^H2	0.01837	0.11510
**Gu-Tyr[b]**	cation-π	0.00581	0.01535	**Gu-Tyr[c]**	cation-π	0.00675	0.02034
	N^…^H	0.00606	0.01805			0.00695	0.02257
	O1^…^H1	0.03793	0.13075				
	O2^…^H2	0.03164	0.11321				

**Figure 3:**
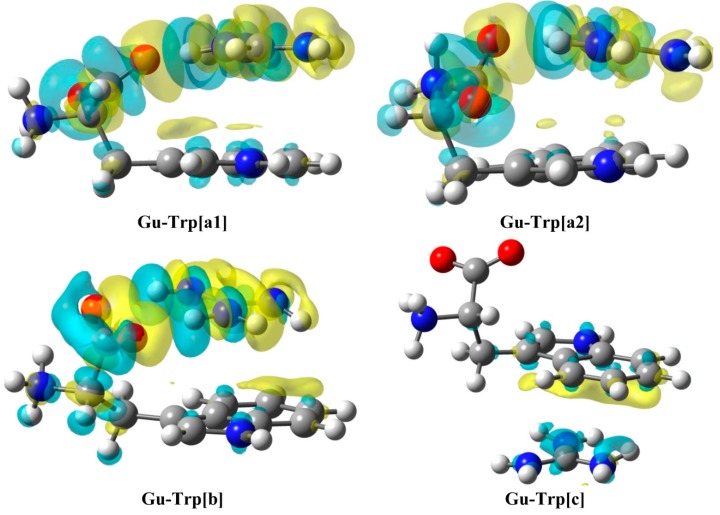
Electron Density Shifts at 0.00045 a.u. of **Gu-Tyr** complexes at the M06-2X/6-311++G(d,p) computational level in PCM–water. Yellow and blue areas represent positive (increase) and negative (decrease) electron density regions respectively.

To achieve a visual description of the electron density changes that result from the complexation process, electron density shift maps (EDS) [35] have been calculated for the complexes **Gu-Trp[a1]**, **Gu-Trp[a2]**, **Gu-Trp[b]**, and **Gu-Trp[c]** (Figure 3).

As observed for [**a**] and [**b**] complexes, a distinctive picture for the electronic density shift is obtained for HBs, *i.e.*, a yellow area (positive) appears between the H and O atoms, which accounts for an increment of the electron density between those two atoms. Besides, the maps corresponding to the cation-π interaction show a charge-gaining region (yellow) between both moieties while a charge-depleting area (light blue) is obtained on top of the amino acids aromatic rings (complexes [**a**] and [**b**]) or the guanidinium cation (complex [**c**]), which evidences the cation-π interaction.

### 2.3. Natural Bond Orbital (NBO) Analysis

The NBO analysis [36] of all the complexes studied has been carried out to assess the orbital interactions established among the monomers and the corresponding second order orbital energies [E(2), kJ·mol^−1^] are presented in Table 3.

For the HB interactions within all the complexes, those of type **[b]** showed the largest E(2) values. These complexes involve two HBs between two heteroatoms interacting with the guanidinium as opposed to the bifurcated HBs interacting with one heteroatom observed in complexes of type **[a]**. The strongest E(2) values are observed in type **[b]** complexes and correspond to a donation from the carbonyl O lone-pairs to the N-H antibonding orbitals of guanidinium (between 56.7–116.9 kJ·mol^−1^). Comparing the E(2) values obtained for the different types of HBs within each complex it can be seen that, in general, the difference between both values is bigger for complexes with bifurcated HBs, finding the biggest difference for type **[a2]** complexes.

**Table 3:** Orbital energy [E(2), kJ·mol^−1^] of the complexes studied at the M06-2X/6-311++G(d,p) computational level in PCM–water.

Complex	Orbital Interaction	E(2)	Complex	Orbital Interaction	E(2)
**Gu-Phe[a1]**	BD CC → LP* C_G_	7.2	**Gu-Phe[a2]**	LP C_G_ → BD* CC	4.4
	BD CC → BD* N_G_H	1.6		LP O → BD* NH	70.4
	LP O → BD* NH	63.3		LP O → BD* NHʹ	19.4
	LP O → BD* NHʹ	49.5			
**Gu-Phe[b]**	BD CC → LP* C_G_	3.9	**Gu-Phe[c]**	LP C_G_ → BD* CC	2.4
	LP O → BD* NH	83.7			
	LP Oʹ → BD* NHʹ	74.8			
**Gu-His[a1]**	BD NC_G_ → BD* NC	1.3	**Gu-His[a2]**	LP C_G_ → BD* CC	1.0
	LP O → BD* NH	64.8		LP O → BD* NH	64.6
	LP O → BD* NHʹ	43.8		LP O → BD* NHʹ	15.0
**Gu-His[b]**	LP N_G_ → BD* NH	6.0	**Gu-His[c]**	BD CN_G_ → BD* NH	2.3
	LP O → BD* NH	104.2			
	LP Oʹ → BD* NHʹ	89.4			
**Gu-Trp[a1]**	LP N → LP* C_G_	4.7	**Gu-Trp[a2]**	LP N_G_ → BD* CC	5.6
	BD CC→ BD* NC_G_	4.7		LP C_G_ → BD* CC	4.3
	LP O → BD* NH	41.2		LP O → BD* NH	45.1
	LP O → BD* NHʹ	34.3		LP O → BD* NHʹ	33.8
**Gu-Trp[b]**	LP N_G_ → BD* CC	4.6	**Gu-Trp[c]**	LP N_G_ → BD* NH	2.9
	LP C_G_ → BD* CC	1.1		LP C_G_ → BD* NH	1.4
	LP O → BD* NH	57.4			
	LP Oʹ → BD* NHʹ	56.7			
**Gu-Tyr[a1]**	LP C_G_ → BD* CC	3.7	**Gu-Tyr[a2]**	LP C_G_ → BD* CC	1.6
	LP O → BD* NH_G_	5.6		LP N_G_ → BD* CC	1.5
	LP O → BD* NH	58.0		LP O → BD* NH_G_	9.9
	LP O → BD* NHʹ	52.8		LP N_G_ → BD* NH	6.1
				LP O → BD* NH	89.6
				LP O → BD* NHʹ	3.3
**Gu-Tyr[b]**	LP C_G_ → BD* NH	3.0	**Gu-Tyr[c]**	LP N_G_ → BD* NH	1.0
	LP N_G_ → BD* NH	2.2		LP C_G_ → BD* NH	0.7
	LP O → BD* NH	116.9			
	LP Oʹ → BD* NHʹ	68.6			

In the cation-π interactions the most important orbital exchanges are those from the C-C bonding orbitals of the aromatic systems to an “empty” lone pair of the guanidinium central C atom (BD CC → LP* C_G_) indicating a donation from the aromatic system to the guanidinium. However, also, when an acceptor orbital is appropriately positioned, donation from a guanidinium N or C lone pair to antibonding orbitals on the amino acids occurs. It is important to highlight that the magnitudes of the second order perturbation energies are smaller in cation-π interactions than in HB interactions.

In spite of the slight differences obtained between E(2) and E_i_ values for the different complexes, if a comparison between both is to be made, a good exponential correlation can be found [E(2) = 0.848 e^−0.08(Ei)^, R^2^ = 0.823, see Supporting Information, Figure S4).

### 2.4. Effect on the Aromaticity

In order to study the effect that guanidinium complexation has on the aromaticity of the amino acids in all the systems considered, we have calculated the NICS values at 0, 1 and 2 Å over the ring center of each aromatic system. Some authors prefer the use of NICS(zz) [also called NICS(out-of-plane), zz denomination corresponds to the out-of-plane component in a planar ring system in which the molecular plane is contained into the XY plane] component to describe aromaticity or antiaromaticity. It is known that in some cases, NICS can diagnose delocalization and NICS(zz) predicts the opposite behavior or vice versa; also it has been shown that this occurs mainly because NICS values are contaminated by the in-plane contributions [37,38]. However, and following our previous experience [39,40], the average NICS values have been used for the discussion instead of the NICS(zz) component. Since we want to assess how the aromaticity on the amino acids is affected upon complexation, we have calculated the NICS values of all amino acids as well as of those of the complexes with the same level of theory. All calculated NICS values obtained at 0, 1, and 2 Å have been gathered in Table 4. For comparison purposes, the benzene NICS values calculated at the same level of theory have been included. As it was observed previously[41], the NICS(0) may lead to a non-reliable interpretation of the aromatic properties, since the proximity of the atom nuclei could distort the NICS values.

**Table 4:** NICS values (ppm) for all cation-π interactions complexes studied at the M06-2X/6-311++G(d,p) computational level in PCM-water. In parenthesis are the values for five member ring for **Trp** and its complexes.

Complex	NICS(0)	NICS(1)	NICS(2)
**Benzene**	−7.5	−10.5	−5.2
**Phe**	−7.5	−10.4	−5.1
**Gu-Phe[a1]**	−7.4	−10.3	−5.2
**Gu-Phe[a2]**	−8.5	−10.9	−5.1
**Gu-Phe[b]**	−7.6	−10.1	−5.1
**Gu-Phe[c]**	−7.9	−10.8	−5.2
**His**	−11.5	−10.1	−4.1
**Gu-His[a1]**	−12.5	−10.9	−4.4
**Gu-His[a2]**	−12.2	−10.9	−4.6
**Gu-His[b]**	−12.3	−10.7	−1.4
**Gu-His[c]**	−12.0	−10.4	−4.2
**Trp**	−9.0 (−12.3)	−10.9 (−10.6)	−5.3 (−4.3)
**Gu-Trp[a1]**	−9.6 (−12.9)	−11.0 (−10.5)	−5.6 (−4.6)
**Gu-Trp[a2]**	−9.0 (−12.7)	−11.0 (−10.3)	−5.5 (−4.6)
**Gu-Trp[b]**	−9.4 (−13.1)	−10.9 (−10.8)	−5.4 (−4.8)
**Gu-Trp[c]**	−9.3 (−13.0)	−10.8 (−10.9)	−5.3 (−4.9)
**Tyr**	−8.7	−10.0	−4.8
**Gu-Tyr[a1]**	−8.5	−10.0	−4.8
**Gu-Tyr[a2]**	−8.8	−9.8	−4.9
**Gu-Tyr[b]**	−9.8	−10.4	−4.6
**Gu-Tyr[c]**	−9.2	−10.4	−5.0

The results indicate that, as expected, all amino acids under study are aromatic, with NICS values very close to those of benzene. When the complexes are formed no significant variance in the aromaticity has been found. However, some slight increase of the NICS values for type [**b**] complexes for all amino acids is observed, with the exception of **Trp**; actually, the NICS values for complexes formed with this amino acid remain practically constant. In contrast, it is important to note that for **His** all complexes formed show an increase slightly larger in absolute value than for the other amino acids.

**Figure 4:**
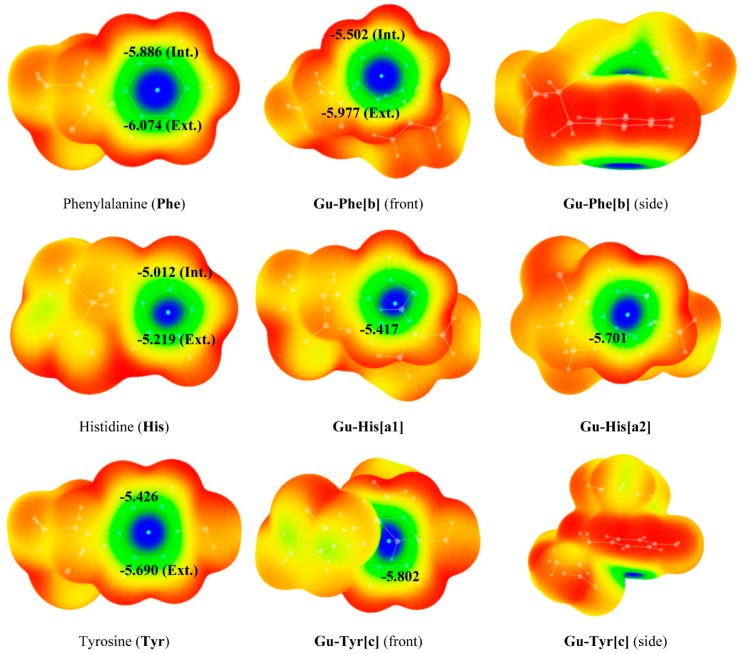
3D-Representation of the NICS values (ppm) on the 0.001 a.u. electron density isosurfaces of **Phe**, **His** and **Tyr** and of the cation-π guanidinium complexes studied at M06-2X/6-311+G(d,p) computational level. Color code: blue < −4.0, Green > −4.0, Yellow > −2.0, Red > 0.0. NICS minima values in the aromatic ring are marked with cyan dots.

In order to obtain additional information on the aromaticity of these systems, we have calculated and plotted the NICS values on the 0.001 a.u. electron density isosurface (resembling the van der Waals surface) for the amino acids selected and all the complexes studied in this work (Figure 4). Comparing the isosurfaces of the isolated amino acids with those of aromatic systems previously studied by us (benzene or pyridine) [41] it can be observed that their aromatic characterizations are similar to those of systems such as benzene or pyridine.

Minima NICS values on the 0.001 a.u. electron density isosurface for the complexes (Figure 4), localized in the center of the rings, reveal that upon complexation, the NICS distribution in the surfaces remains very similar to that in the isolated monomers. However, in the case of Phe minor changes are observed; thus, a minor decrease in the aromaticity is detected for the **Gu-Phe[b]** complex, while for complexes **Gu-His[a1]**, **Gu-His[a2]** and **Gu-Tyr[c]** an slight increase is observed upon complexation.

## 3. Experimental Section

All systems (monomers and complexes) have been optimized using the M06-2X [24] computational level with the 6-311++G(d,p) [25] basis sets. The M06-2X functional has been shown to properly describe weak interactions taking into account dispersion forces where other traditional functionals fail.

Effects of water solvation have been included by means of the SCFR-PCM approaches implemented in the Gaussian09 package [26] including dispersing, repulsing, and cavitating energy terms of the solvent starting from the gas-phase geometries and re-optimizing.

The interaction energy of the complexes has been calculated as the difference between the energy of the supermolecule (complex) and the sum of the energies of the isolated monomers in their minimum energy configuration.

The electron density of the complexes has been analyzed within the Atoms in Molecules (AIM) [27] theory using AIMAll software [28]. The Natural Bond Orbital (NBO) [36] method has been used to analyze the interaction of the occupied and unoccupied orbitals with the NBO-3 program [42], since this kind of interaction is of utmost importance in the formation of hydrogen bonds and other charge transfer complexes.

The theoretical NICS values were calculated using the GIAO method on the optimized geometries. To calculate the spatial distribution of the NICS, its values have been calculated on a three-dimensional (3D) cubic grid of 12 Å side following the procedure described by Sánchez-Sanz *et al.* [41]. The points in the grid are located at 0.2 Å from each other in the three spatial directions. The result is a cube with 226,981 NICS values that, in the next step, were represented within the electron density isosurface of 0.001 a.u. using the WFA program [43].

The intramolecular electron density shift (EDS) has been obtained using the fragmentation scheme proposed in ref 33. This method proposes the calculation of the EDS of the intramolecular interaction by comparing the electron density of the interacting moieties. The EDS is calculated using Equation (1):

EDS = ρ(complex) − ρ(amino acid) − ρ(guanidinium)
(1)

## 4. Conclusions

The complexes established by the guanidinium cation and the aromatic amino acids, **Phe**, **His**, **Trp** and **Tyr** by means of cation-π and hydrogen bonding interactions have been computationally studied using PCM–water at the M06-2X/6-311++G(d,p) level.

All the minima found correspond to complexes of three different types: complexes type **[a]** formed by a bifurcated HB between two guanidinium H atoms and one O atom of the carboxylic group belonging to the amino acid and a cation-π interaction between both monomers; complexes type **[b]** formed by a double parallel HB interaction of two guanidinium H atoms with two O of the carboxylic group of the amino acid plus a cation-π interaction; and complexes type **[c]** formed only by cation-π interactions between the aromatic moiety of the amino acids and guanidinium.

The computed interaction energies show that the most stable complexes found for all the aromatic amino acids were the complexes type **[a]** and **[b]**, which exhibit both HB and cation-π interactions.

The AIM analysis of these guanidinium-amino acids complexes showed a number of interactions (HBs and cation-π) established between both moieties as shown by the bond critical points found. The electron density at the BCPs and its Laplacian are in agreement with weak interactions, being the parallel HBs of the complexes type **[b]** stronger than the bifurcated ones found in type **[a]** complexes. Different correlations have been found between the interatomic distances and the value of the electron density at the BCP for the HB interactions.

Natural Bond Orbital analysis has been performed allowing a better understanding of the nature of the interactions. Based on the perturbation energy E(2), the most important bonding interactions were the HBs found in complexes **[a]** and **[b]** from the lone pair of the oxygen in an amino acid to an antibonding N-H orbital of guanidinium (LP O → BD* NH). Further, cation-π interactions have been found in almost all complexes being the most important bonding contribution that corresponding to the interaction between a bonding C-C or C-N orbitals and the empty ‘lone pair’ of the guanidinium central C. In all these complexes, an exponential correlation was found between the E(2) and the interaction energy E_i_.

Finally, to understand the effect that the complexation with a guanidinium cation has on the aromaticity of the amino acid studied, the NICS values were calculated and the 3D NICS surfaces were produced, finding that the aromatic character was not heavily modified upon complexation. All this information indicates that guanidinium containing compounds would positively interact with protein binding sites rich in aromatic amino acids without modifying the properties of those residues.

## Supplementary Materials

Supplementary materials can be accessed at: http://www.mdpi.com/1420-3049/20/05/9214/s1.
