# Supplementary material for: Aromatic Amino Acids-Guanidinium Complexes through Cation-π Interactions
Source: Molecules. 2015 May 20;20(5):9214–28. doi: 10.3390/molecules20059214 (PMC6272432; doi:10.3390/molecules20059214)
Supplement: Supplementary file 1 [file molecules-20-09214-s001.pdf]

Supporting Information

Figure S1. Molecular graph (AIM) for all the complexes in PCM-Water.

Figure S2. Exponential relationship between the interatomic distances (Å) and the electron density at the BCPs for the HB interactions.

Figure S3. Exponential relationship between the interatomic distances (Å) and the Laplacian of the electron density at the BCPs for the cation-π interactions.

Figure S4. Exponential correlation found between the interaction energies, E_i ($kJ \cdot mol^{-1}$) and NBO second order perturbation energy E(2) ($kJ \cdot mol^{-1}$).

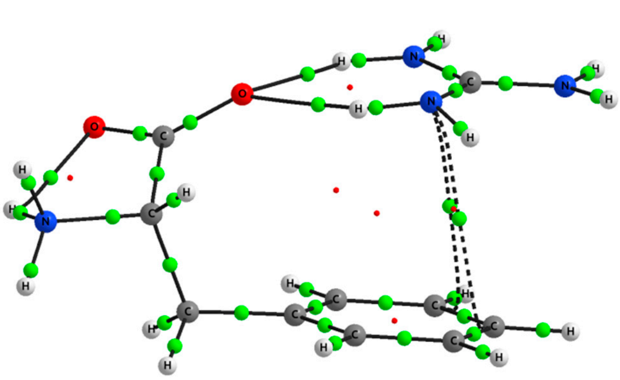

Gu-Phe[a1]

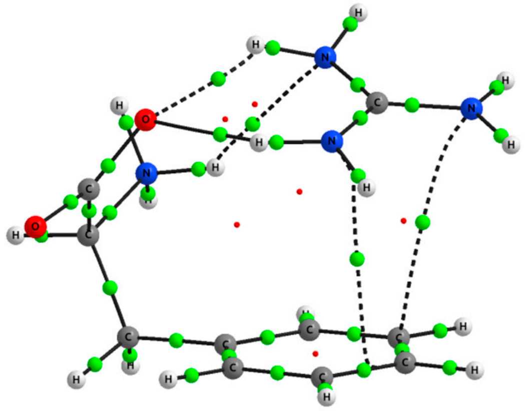

Gu-Phe[a2]

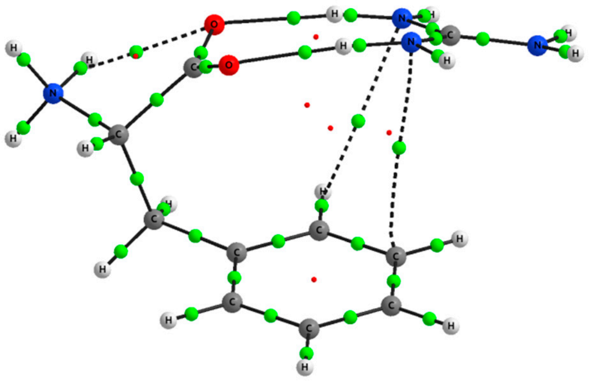

Gu-Phe[b]

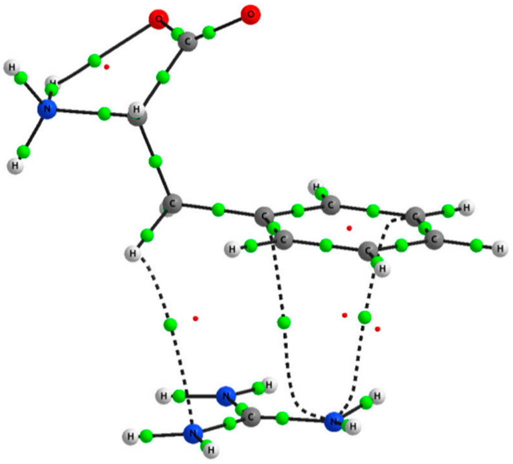

Gu-Phe[c]

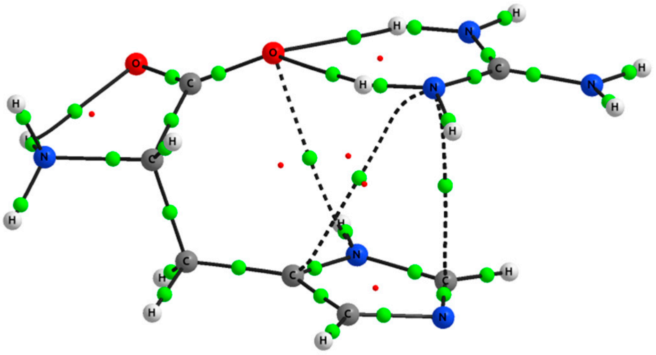

Gu-His[a1]

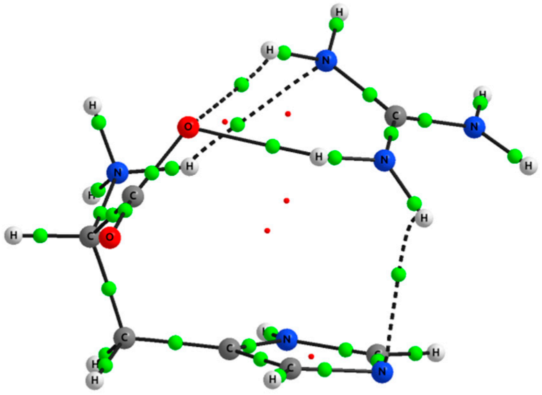

Gu-His[a2]

Figure S1. *Cont.*

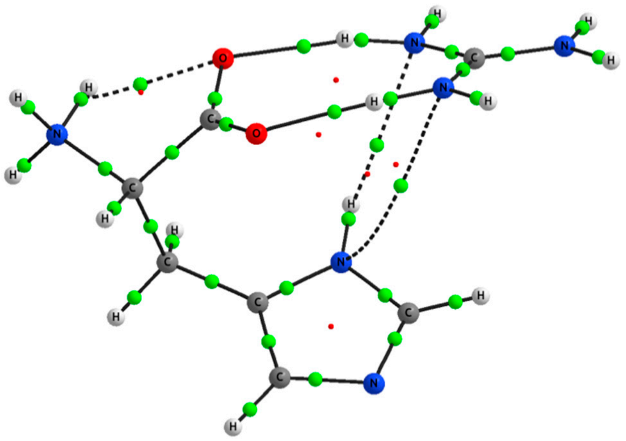

Gu-His[b]

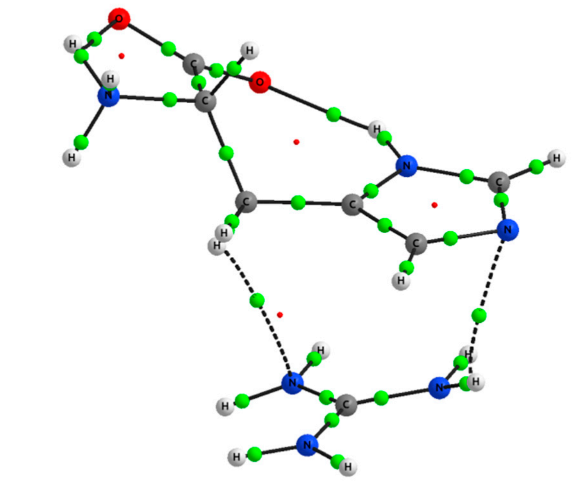

Gu-His[c]

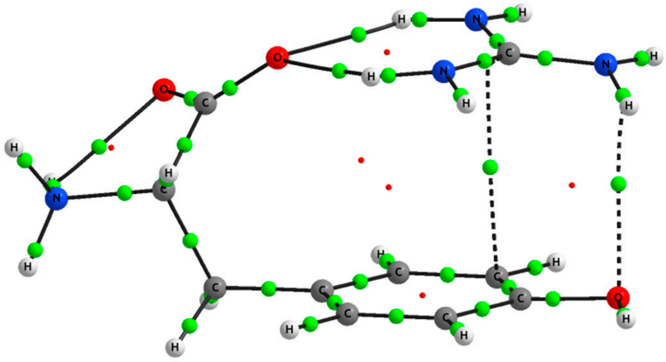

Gu-Tyr[a1]

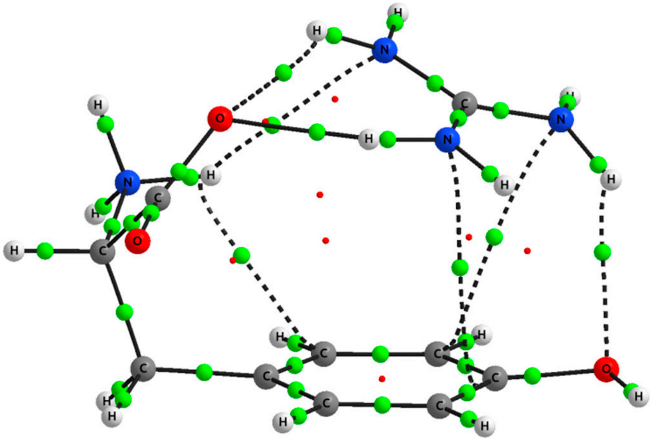

Gu-Tyr[a2]

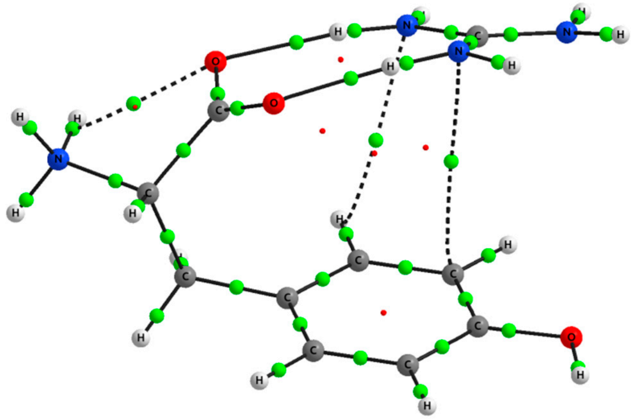

Gu-Tyr[b]

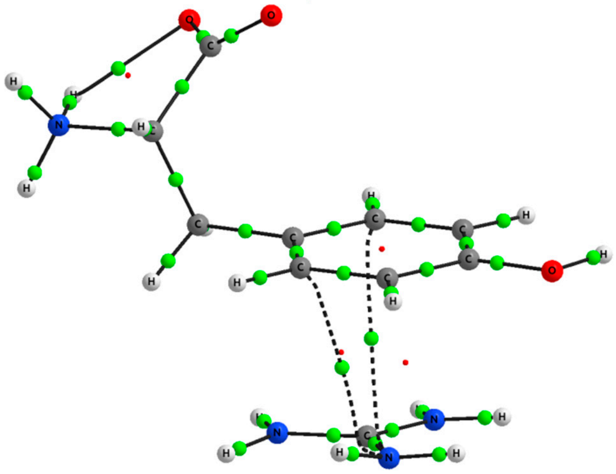

Gu-Tyr[c]

Figure S1. Molecular graph (AIM) of the complexes calculated at the M06-2X/6-311++G(d,p) computational level in PCM-water phase. Green and red balls indicate bond and ring critical points, respectively.

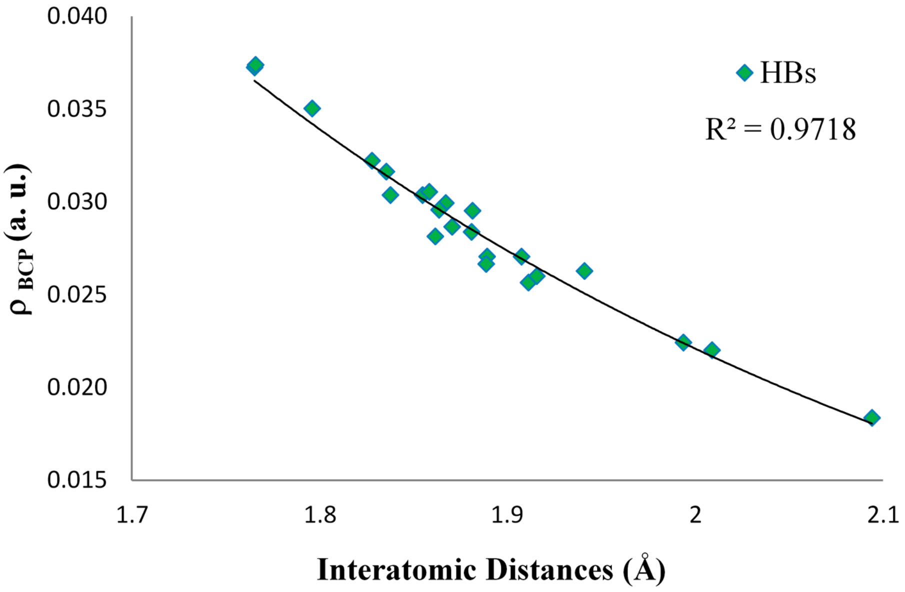

Figure S2. Exponential relationship found between the interatomic distances (Å) and the value of the electron density at the BCPs for the HB interactions.

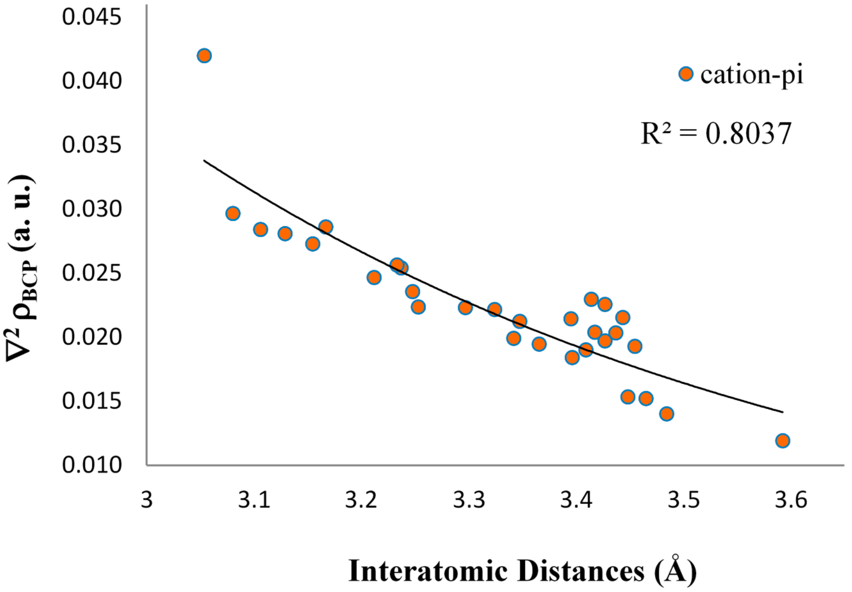

Figure S3. Exponential relationship found between the interatomic distances (Å) and the value of the Laplacian of the electron density at the BCPs for the cation-π interactions.

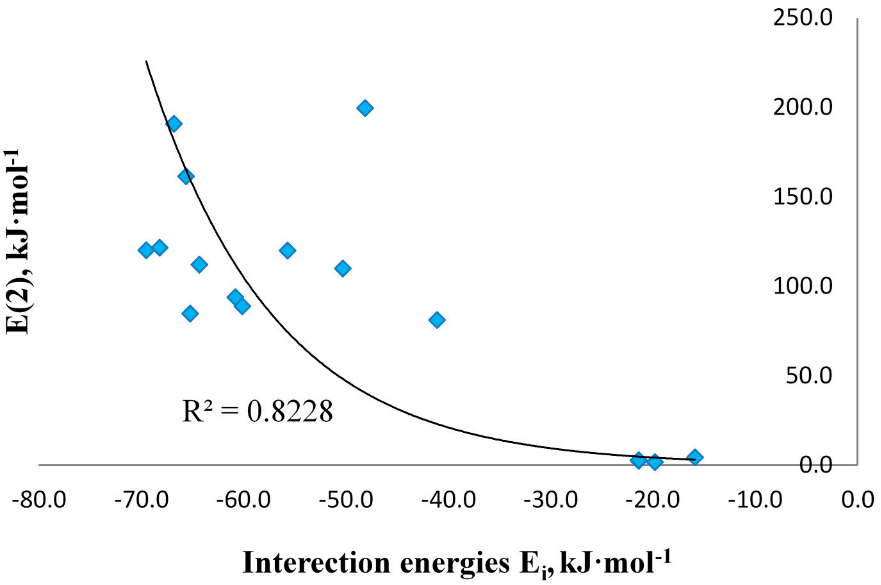

Figure S4. Exponential **c**orrelation found between the interaction energies, E_i (kJ·mol^{-1}) and NBO second order perturbation energy E(2) (kJ·mol^{-1}).
